# Supplementary material for: The Systems Biology Research Tool: evolvable open-source software
Source: BMC Syst Biol. 2008 Jun 29;2:55. doi: 10.1186/1752-0509-2-55 (PMC2446383; doi:10.1186/1752-0509-2-55)
Supplement: Additional file 1 — SBRT Archive. An archive of the current version of the Systems Biology Research Tool. [file 1752-0509-2-55-S1.zip › sbrt-1.4.0/doc/developers_guide/api/sbrt/shell/text/math/LinearCombFormatV2.html]

LinearCombFormatV2


|  |  |  |
| --- | --- | --- |
| **Overview** \| **Package** \| **Class** \| **Use** \| **Tree** \| **Deprecated** \| **Index** \| **Help** | |  |
| **PREV CLASS**   **NEXT CLASS** | **FRAMES**    **NO FRAMES**     **All Classes** |  |
| SUMMARY: NESTED \| FIELD \| CONSTR \| METHOD | DETAIL: FIELD \| CONSTR \| METHOD |  |


---


## sbrt.shell.text.math Class LinearCombFormatV2<V>

```
java.lang.Object
  sbrt.shell.text.math.LinearCombFormatV2<V>
```

**Type Parameters:**: `V` - the variable type.

**All Implemented Interfaces:**: Format, Formatter<LinearComb<V>>, LinearCombFormat<V>, MathExprFormat<LinearComb<V>,V>, Parser<LinearComb<V>>, SimpleFormat<LinearComb<V>>

---

``` public final class LinearCombFormatV2<V> extends java.lang.Object implements LinearCombFormat<V> ```

This class is a concrete implementation of `LinearCombFormat`. The
linear combination format defined by this class looks like:

*coefficient_1_ variable_1_ + constant*

In terms, the
coefficients must appear before the variable, and the two must be separated
by at least one whitespace character. Terms must be separated by `+`
signs. Coefficients, constants, variables, and `+` signs must all be
separated from each other by at least one whitespace character, which means
variable strings cannot contain any whitespace characters. The constant in a
linear combination string can occurr in any sensible position.

**Author:**
:   This class was written and documented by Jeremiah Wright while in the
    Wagner lab.

---

**Constructor Summary**

`LinearCombFormatV2(SimpleFormat<V> variableFormat)`
  
          Constructs a new linear combination format using the provided format.

`LinearCombFormatV2(SimpleFormat<V> variableFormat,
double zeroCutoff)`
  
          Constructs a new linear combination format using the provided arguments.


| **Method Summary** | |
| --- | --- |
| `java.lang.String` | `format(LinearComb<V> expression)`             Returns a formatted string representation of the provided linear combination. |
| `static LinearCombFormatV2<java.lang.String>` | `getBasic()`             Returns a `LinearCombFormatV1` that does not format or parse the variables supplied to it. |
| `CoeffFormatV2` | `getCoeffFormat()`             Returns the format used for coefficients. |
| `LinCombTermFormatV2<V>` | `getTermFormat()`             Returns the format used for terms. |
| `SimpleFormat<V>` | `getVariableFormat()`             Returns the format used for variables. |
| `LinearComb<V>` | `parse(java.lang.String expression)`             Parses the provided string and returns its corresponding linear combination. |

**Methods inherited from class java.lang.Object**

`clone, equals, finalize, getClass, hashCode, notify, notifyAll, toString, wait, wait, wait`

**Methods inherited from interface sbrt.shell.text.Formatter**

`format`

**Constructor Detail**

### LinearCombFormatV2

```
public LinearCombFormatV2(SimpleFormat<V> variableFormat,
                          double zeroCutoff)
```

:   Constructs a new linear combination format using the provided arguments.

    **Parameters:**: `variableFormat` - the format to use for variables.: `zeroCutoff` - the zero cutoff for constants and coefficients. **See Also:**: `LinearComb.getZeroCutoff()`

---


### LinearCombFormatV2

```
public LinearCombFormatV2(SimpleFormat<V> variableFormat)
```

:   Constructs a new linear combination format using the provided format. The
    zero cutoff is set to 0.0d.

    **Parameters:**: `variableFormat` - the format to use for variables. **See Also:**: `LinearComb.getZeroCutoff()`


**Method Detail**

### getBasic

```
public static LinearCombFormatV2<java.lang.String> getBasic()
```

:   Returns a `LinearCombFormatV1` that does not
    format or parse the variables supplied to it. In other
    words, it returns a `LinearCombFormatV1` with
    the most basic possible functionality. Empty strings are not
    allowed however.

    :   **Returns:**: a `LinearCombFormatV1` that does not format or parse the variables supplied to it.

---


### getVariableFormat

```
public SimpleFormat<V> getVariableFormat()
```

:   Returns the format used for variables.

    :   **Returns:**: the format used for variables.

---


### getTermFormat

```
public LinCombTermFormatV2<V> getTermFormat()
```

:   Returns the format used for terms.

    :   **Returns:**: the format used for terms.

---


### getCoeffFormat

```
public CoeffFormatV2 getCoeffFormat()
```

:   Returns the format used for coefficients.

    :   **Returns:**: the format used for coefficients.

---


### format

```
public java.lang.String format(LinearComb<V> expression)
```

:   Returns a formatted string representation of the provided linear
    combination.

    :   **Specified by:**: `format` in interface `LinearCombFormat<V>` **Specified by:**: `format` in interface `MathExprFormat<LinearComb<V>,V>`
    :   **Parameters:**: `expression` - the linear combination to be formatted. **Returns:**: a formatted string representation of the provided linear combination.

---


### parse

```
public LinearComb<V> parse(java.lang.String expression)
```

:   Parses the provided string and returns its corresponding linear
    combination.

    :   **Specified by:**: `parse` in interface `LinearCombFormat<V>` **Specified by:**: `parse` in interface `MathExprFormat<LinearComb<V>,V>` **Specified by:**: `parse` in interface `Parser<LinearComb<V>>`
    :   **Parameters:**: `expression` - the string to be parsed. **Returns:**: the linear combination corresponding to the provided string.


---


|  |  |  |
| --- | --- | --- |
| **Overview** \| **Package** \| **Class** \| **Use** \| **Tree** \| **Deprecated** \| **Index** \| **Help** | |  |
| **PREV CLASS**   **NEXT CLASS** | **FRAMES**    **NO FRAMES**     **All Classes** |  |
| SUMMARY: NESTED \| FIELD \| CONSTR \| METHOD | DETAIL: FIELD \| CONSTR \| METHOD |  |


---
